# Low-level viraemia despite emergence of dolutegravir-resistant variants

**DOI:** 10.4102/sajhivmed.v23i1.1398

**Published:** 2022-09-30

**Authors:** Johannes C. Botha, Kim Steegen, Mariam Edoo, Jeremy Nel, Gert U. van Zyl

**Affiliations:** 1 Department of Medical Virology, Faculty of Medicine and Health Sciences, Stellenbosch University, Cape Town, South Africa; 2 Department of Molecular Medicine and Haematology, Faculty of Health Sciences, National Health Laboratory Service, Johannesburg, South Africa; 3 Department of Molecular Medicine and Haematology, Faculty of Health Sciences, University of the Witwatersrand, Johannesburg, South Africa; 4 Department of Medicine, Helen Joseph Hospital, Johannesburg, South Africa; 5 Department of Medicine, University of the Witwatersrand, Johannesburg, South Africa; 6 Department of Molecular Medicine, National Health Laboratory Service, Cape Town, South Africa

## Introduction

Dolutegravir (DTG), an integrase strand transfer inhibitor (INSTI)-based HIV-1 therapy, is widely recommended in first-line and second-line regimens.^1,2^ Integrase strand transfer inhibitor resistance mutations associated with DTG-containing regimens have been well described, most often occurring after DTG monotherapy or in INSTI-experienced patients.^3^Although rare, emergence of these mutations has also been described in patients on DTG-containing triple-drug regimens^4^ and INSTI-naïve patients.^5,6^ The R263K mutation is commonly associated with the emergence of DTG resistance but reduces viral fitness and DNA integration.^7,8^ Here we describe a case of very slow viral decline (~42 months) in a treatment-experienced, INSTI-naïve patient on a DTG-based triple therapy regimen.

### Case

**FIGURE 1:**
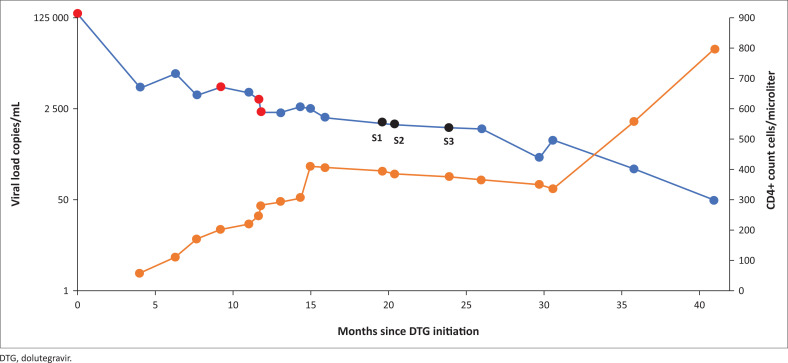
Patient viral load (blue) and CD4 data (orange) graph displayed in months since dolutegravir initiation indicating samples available for further investigation (black dots) (S1, S2 & S3). Routine drug resistance testing performed on multiple occasions are indicated by red dots.

A 43-year-old man was diagnosed with HIV-1 in 2012 and commenced on an antiretroviral treatment (ART) regimen consisting of tenofovir disoproxil fumarate (TDF), emtricitabine (FTC) and efavirenz (EFV). Initial poor adherence to treatment and virologic failure was reported for 2012–2015 (viral load range 14 269 copies/mL – 173 455 copies/mL), and due to concerns of having possibly acquired resistance to this regimen, treatment was empirically switched according to national guidelines to lamivudine (3TC), zidovudine (AZT) and lopinavir/ritonavir (LPV/r). He remained on this regimen with his family practitioner from 2015 until early 2018, when he was referred back to our hospital for persistently high HIV viral loads. He admitted to poor adherence secondary to LPV/r-associated diarrhoea, and was subsequently switched to 3TC, AZT and DTG. Patient clinical data and treatment history are shown in Table 1. After initial viral load decline to 6500 copies/mL the viral load returned to 11 700 copies/mL and non-adherence was suspected. Although admitting poor adherence during 2012–2018 the patient claimed perfect adherence since starting his DTG regimen. Adherence was confirmed initially by means of directly observed therapy, and then by unannounced random (same-day unscheduled clinic visit requests) 3TC and DTG drug concentration testing on two and four occasions (3TC 1.24 μg/mL – 4.45 μg/mL, DTG 0.583 μg/mL – 1.354 μg/mL in plasma). Drug resistance testing on multiple occasions (Figure 1) identified M184V which, results in resistance to 3TC and increases susceptibility to AZT; K65R was also detected and also increases AZT susceptibility.^9^ Integrase sequencing indicated susceptibility to DTG from January 2018 until September 2019 (21 months), after which resistance to DTG (N155H and R263K) was detected (January 2020) in a single sample. Follow-up INSTI drug resistance testing could not confirm DTG resistance. Even though DTG resistance was detected at one time point, the viral load continued to decline (Figure 1) despite the patient remaining on a DTG-based regimen. Plasma samples from three time points and cells (buffy coat/peripheral blood mononuclear cells [PBMCs]) from two time points were collected (S1, S2 & S3) for further investigation (Table 1 and Figure 1). All samples used in this study were collected after DTG initiation, with the relevant regimen over sampling period S1 and S2 being 3TC/AZT/DRV/r and S3 being 3TC/AZT/DTG.

**TABLE 1:** Patient clinical data and treatment regimens.

Variable	Description
Age	43
Gender	Male
Diagnosis	2012
Samples (time after DTG initiation) and viral load (copies/mL)	S1 (~16 months): 1420
	S2 (~20 months): 1290
	S3 (~24 months): 1128
Duration of detectable viraemia	~42 months
Previous ART regimens and viral load range (copies/mL)	FTC/TDF/EFV (2012–2015): 29 271–173 455
	3TC/AZT/LPV/r (2015–2018): 14 269–149 000
Study period ART regimen and viral load range (copies/mL)	3TC/AZT/DTG (January 2018 – August 2019): 313–149 000
	3TC/AZT/DRV/r (August 2019 – November 2019): 650
	3TC/AZT/DTG (November 2019 onward): 50–193

ART, antiretroviral treatment; TDF, tenofovir disoproxil fumarate; FTC, emtricitabine; EFV, efavirenz; AZT, zidovudine; DRV, darunavir; DTG, dolutegravir; LPV, lopinavir; r, ritonavir; PBMCs, peripheral blood mononuclear cells.

## Molecular workup

PBMCs were isolated from freshly collected whole blood ethylenediaminetetraacetic acid specimens according to the HIV/AIDS Network Coordination Cross-Network Peripheral Blood Mononuclear Cell Processing Standard Operating Procedure (www.hanc.info/labs/labresources/procedures/Pages/pbmcSop.aspx). Cell-associated DNA and RNA were isolated from PBMCs and viral RNA was isolated from plasma as reported by Hong et al.^10^ Single genome amplification products covering the p6-protease-reverse transcriptase (p6PrRT) region were generated and Sanger sequencing was performed as described previously.^11^ Additionally, single genome amplicons covering the entire polymerase region were analysed for drug resistance mutations. Population and single genome nucleotide sequences were analysed for drug resistance mutations.

### Ethical considerations

Ethical approval was obtained from the University of the Witwatersrand, where the patient was enrolled. Written informed consent was obtained from the patient.

## Results

Patient p6PrRT nucleotide sequences from S1 (plasma RNA and buffy coat DNA), S2 (plasma RNA) and S3 (plasma RNA, cell-associated DNA and RNA) indicate drug resistance mutation M184V (Table 2).

**TABLE 2:** Patient p6-protease-reverse transcriptase nucleotide sequences obtained from various sources for each sample with drug resistance mutations detected.

Sample (time after DTG initiation)	Sequence source	Number of sequences obtained	Drug resistance mutations	
			NRTI	NNRTI
S1 (~16 months)	Plasma RNA	8	A62V, K65R, M184V	L100I, K103N
	Buffy coat DNA	24	None	None
S2 (~20 months)	Plasma RNA	12	A62V, K65R, M184V	L100I, K103N
S3 (~24 months)	Plasma RNA	22	A62V, K65R, M184V	L100I, K103N
	PBMC DNA	24	None	None
	PBMC RNA	21	None	K103N

NNRTI, non-nucleoside reverse transcriptase inhibitor; NRTI, nucleoside reverse transcriptase inhibitor; PBMC, peripheral blood mononuclear cell; DTG, dolutegravir.

**FIGURE 2:**
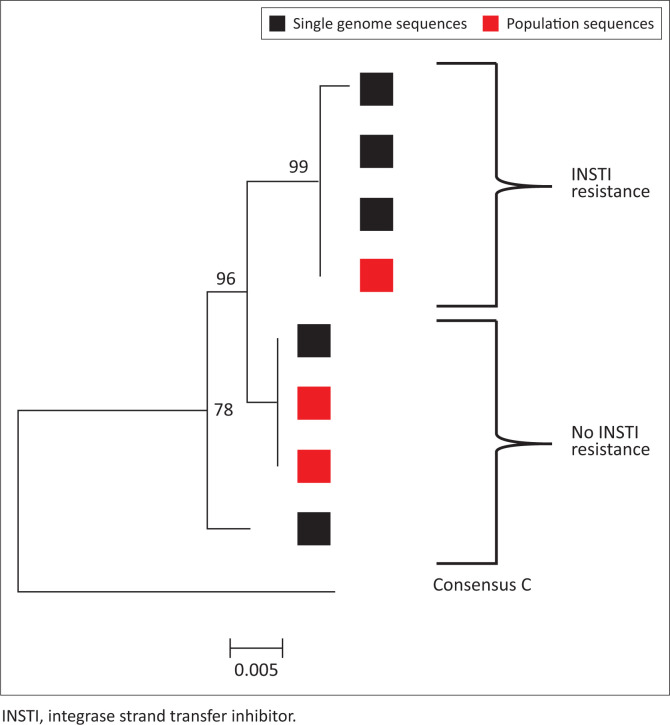
Neighbour joining p-distance phylogenetic tree of integrase nucleotide sequences generated from population and single genome amplicons.

Mutations N155K and R263K were only detected once in S3 and were confirmed with single genome sequencing (Table 3). Drug resistance analyses of integrase population and single genome amplicons from plasma RNA indicate two distinct populations: INSTI resistant and INSTI non-resistant (Table 3). These two integrase populations are reflected in phylogenetic analysis (Figure 2).

**TABLE 3:** Integrase population and single genome sequences indicating associated drug resistance mutations.

Sequence origin	Time since DTG initiation	Genes	INSTI major	INSTI accessory
Routine integrase drug resistance population sequences	12 months	IN	None	None
	~20 months	IN	None	None
	S3 (24 months)	IN	N155H, R263K	D232N
Single genome sequences	S3 Sequence 1 (24 months)	IN	None	None
	S3 Sequence 2 (24 months)	IN	None	None
	S3 Sequence 3 (24 months)	IN	N155H, R263K	D232N
	S3 Sequence 4 (24 months)	IN	N155H, R263K	D232N
	S3 Sequence 5 (24 months)	IN	N155H, R263K	D232N

INSTI, integrase strand transfer inhibitor; DTG, dolutegravir.

At the time INSTI resistance was definitively confirmed, the patient’s HIV viral load had already declined to 50 copies/mL. After much discussion with the patient, the patient elected to continue on his current regimen (rather than switch to a non-DTG-based regimen) with regular HIV viral load monitoring.

## Discussion

We report a patient with viral load suppression despite a mixed viral population that includes INSTI-resistant variants with N155H and R263K. The patient was treatment experienced but INSTI naïve when initiated on a DTG-based regimen. DTG-resistant mutations N155H and R263K were first detected 24 months after DTG initiation; however, despite this the HIV-1 viral load continued decline to 50 copies/mL after 42 months. It is unclear why the DTG-resistant variants did not outcompete DTG-susceptible variants, but the low viral fitness could have contributed. Mutations R263K, M184V and K65R are all associated with decreased viral fitness and here occurred on the same viral variant. The slow but continued viral load decline could be due to the remaining activity and hyper-susceptibility to AZT, in the presence of the M184V and K65R mutations.^12,13,14,15^ Moreover CD4^+^ cell recovery and associated improved cytotoxic T-cell responses could have contributed to immune reconstitution to HIV and resulting viral load decline.

Although previously thought to be rare, several cases of virological failure as a result of DTG resistance in INSTI-naïve patients have been described.^4,16,17^ The factors associated with DTG resistance are poorly understood. Whereas DTG resistance when used in first-line therapy is exceedingly rare, it is not unusual when DTG is administered to treatment-experienced patients,^1,6^ even when previously INSTI naïve. It is interesting that six of the nine cases of treatment-experienced, INSTI-naïve cases in the NADIA study^1^ with drug resistance received the same regimen as this patient, albeit that in our case this was given as a third-line regimen in contrast to NADIA where the patients received DTG in second-line treatment. Reported clinical risk factors associated with emergence of DTG resistance include poor treatment adherence, drug interactions and HIV factors such as a high baseline viral load.^6^ In this case we report good adherence but the high viral load (> 100 000 copies/mL) prior to DTG initiation could be a contributing risk factor, even though INSTI resistance was only detected after 24 months. The combination of N155H and R263K is associated with virologic failure on DTG-based regimens,^4,18,19^ in spite of the fitness cost of R263K.^7^ Even though N155H partially compensates for the R263K viral fitness cost, development of compensatory mutations to the N155H, R263K strain may be unlikely or slow despite drug pressure.^20^ No additional risk factors were identified in this case.

The presence of DTG resistance only partially explains the viraemia in this patient but offers no clarity on the susceptible sub-population. The detectable viraemia could be the result of intermittent adherence or compartmentalised replication or clonal viraemia.^11,21,22^ Random unannounced drug concentration testing on various occasions suggest that intermittent adherence is an unlikely cause of the viraemia. Both compartmentalised replication and clonal viraemia could explain the relatively high viral load for an extended period of time. We investigated clonal viraemia in this case as described by Halvas et al.,^11^ but we were not able to find a matching proviral clone to the monotypic plasma virus population (data not shown); further investigation could be performed to shed light on this.

This case report provides further evidence that DTG is not impervious to drug resistance, especially when used in treatment-experienced patients. It also suggests that patients with DTG resistance may not present with immediate viral rebound due to the fitness cost of mutations and could present with delayed viral load suppression or low-level viraemia. It is not known whether this patient’s HIV viral load will continue to remain low or whether additional compensatory mutations might allow the drug-resistant variant to regain fitness resulting in rebound. Continued vigilance is required when using DTG in treatment-experienced patients and sensitive drug resistance assays may be needed to detect drug resistance at low viral load levels.
